# Novel insight into cGAS-STING pathway in ischemic stroke: from pre- to post-disease

**DOI:** 10.3389/fimmu.2023.1275408

**Published:** 2023-10-17

**Authors:** Xiaoqi Ma, Dan Xin, Ruining She, Danhong Liu, Jinwen Ge, Zhigang Mei

**Affiliations:** ^1^ Key Laboratory of Hunan Province for Integrated Traditional Chinese and Western Medicine on Prevention and Treatment of Cardio-Cerebral Diseases, College of Integrated Traditional Chinese and Western Medicine, Hunan University of Chinese Medicine, Changsha, Hunan, China; ^2^ Institute of Pharmacy, Shandong University of Traditional Chinese Medicine, Jinan, China; ^3^ Hunan Academy of Chinese Medicine, Hunan University of Chinese Medicine, Changsha, China

**Keywords:** cGAS-STING pathway, ischemic stroke, neuroinflammation, microglia, intervention target

## Abstract

Ischemic stroke, a primary cause of disability and the second leading cause of mortality, has emerged as an urgent public health issue. Growing evidence suggests that the Cyclic GMP-AMP synthase (cGAS)- Stimulator of interferon genes (STING) pathway, a component of innate immunity, is closely associated with microglia activation, neuroinflammation, and regulated cell death in ischemic stroke. However, the mechanisms underlying this pathway remain inadequately understood. This article comprehensively reviews the existing literature on the cGAS-STING pathway and its multifaceted relationship with ischemic stroke. Initially, it examines how various risk factors and pre-disease mechanisms such as metabolic dysfunction and senescence (e.g., hypertension, hyperglycemia, hyperlipidemia) affect the cGAS-STING pathway in relation to ischemic stroke. Subsequently, we explore in depth the potential pathophysiological relationship between this pathway and oxidative stress, endoplasmic reticulum stress, neuroinflammation as well as regulated cell death including ferroptosis and PANoptosis following cerebral ischemia injury. Finally, it suggests that intervention targeting the cGAS-STING pathway may serve as promising therapeutic strategies for addressing neuroinflammation associated with ischemic stroke. Taken together, this review concludes that targeting the microglia cGAS-STING pathway may shed light on the exploration of new therapeutic strategies against ischemic stroke.

## Highlights

- The cGAS-STING pathway has emerged as a mediator of inflammation in response to the detection of dsDNA.
- The cGAS-STING pathway is closely associated with the initiation of the neuroinflammatory response in ischemic stroke.
- The cGAS-STING pathway is involved in various forms of RCD, including ferroptosis and PANoptosis.
- The HMGB1-NETs-cGAS-STING pathway in microglia activation may be a promising target for the therapy against hemorrhagic transformation.

## 1 Introduction

Stroke poses a significant global health concern, representing the second leading cause of mortality worldwide, with at least 6 million fatalities and impacting 13 million individuals annually (1, 2). Ischemic stroke is the most common type, accounting for over 85% of cases and exhibiting an increase in annual incidence over the past few years (3). Most stroke incidents are associated with established cerebrovascular risk factors, with metabolic factors such as hypertension, hyperglycemia, and hyperlipidemia contributing to over 80% of cases, as reported by the European Stroke Organization (4). Implementing primary prevention strategies, such as the timely management of blood pressure (BP), lipids, and glucose to regulate associated risk factors, can significantly enhance patient survival rates and optimize treatment and outcomes of this patient population.

The pathogenesis of ischemic stroke is multifaceted, involving intricate pathological mechanisms. Disrupted cerebral blood flow results in inadequate ATP and nutrient supply, instigating a cascade of pathophysiological events such as oxidative stress, endoplasmic reticulum (ER) stress, and neuroinflammatory responses, culminating in neuronal demise and dysfunction (5). Thrombolytic therapy with tissue-type plasminogen activator (t-PA) has been established as the standard of care treatment for moderate to severe ischemic stroke (6). The narrow therapeutic window and the risk of hemorrhagic transformation (HT) often lead to unsatisfactory clinical outcomes and prognosis in cases of ischemic stroke (7). Therefore, it is imperative to identify strategies to intervene in the pathological mechanisms associated with ischemic stroke and mitigate the adverse effects of thrombolytic therapy. It is well-established that the inflammatory response plays a substantial role in stroke outcomes, such as stroke incidence, pathology, and the efficacy of thrombolytic therapy. Therefore, it is crucial to comprehensively modulate the inflammatory response to improve stroke injury management. The cGAS-STING pathway has demonstrated significant potential in this regard.

**Figure 1:**
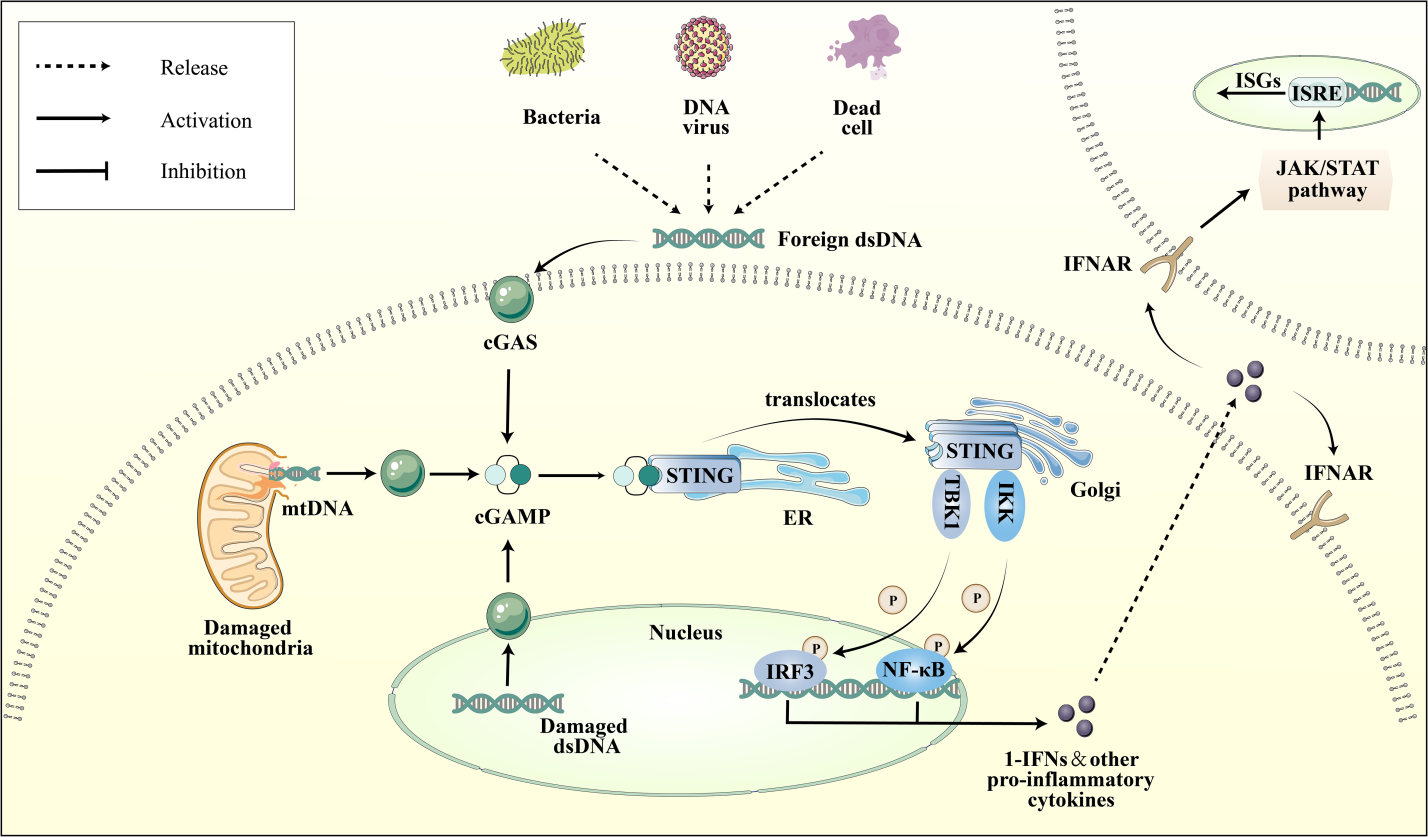
Depiction of the cGAS-STING pathway. Various dsDNA molecules derived from viruses, bacteria, deceased cells, or cellular organelles such as the nucleus and mitochondria, bind to and activate the corresponding sites of the dsDNA sensor cGAS. Subsequently, cGAS catalyzes the synthesis of 2′3′-cGAMP, which binds to the ER adaptor STING. Upon activation, STING translocates from the ER to the Golgi apparatus, where it activates the kinases TBK1 and IKK. These kinases phosphorylate IRF3 and NF-κB, respectively. IRF3 and NF-κB then transcribe pro-inflammatory cytokines in the nucleus, which are subsequently released to activate responsive receptors and initiate an inflammatory cascade response, such as the IFNR-mediated JAK-STAT signal pathway. Ultimately, it regulates the ISRE, thereby promoting the expression of ISGs.

The cGAS-STING pathway is an integral part of the innate immune system, with cGAS acting as a pattern recognition receptor (PRR) that is response to cellular stress or pathogenic invasion (8). PRRs are pivotal in detecting pathogen-associated molecular patterns (PAMPs) and damage-associated molecular patterns (DAMPs) (9). cGAS can directly sense DAMPs, including mislocalized host double-stranded DNA (dsDNA) as well as pathogen-derived dsDNA from PAMPs, such as bacterial and viral (10). Interacting with dsDNA, cGAS synthesizes the second messenger cyclic GMP-AMP (cGAMP). Upon the binding of cGAMP to STING, there is a rearrangement of the STING ligand-binding domain, transitioning it from an open to a closed conformation. This results in the formation of an activated STING tetramer (11), leading to its translocation from the ER to Golgi apparatus. Then STING binds and activates the TANK-binding kinase 1 (TBK1) and Interferon Regulatory Factor 3 (IRF-3) (12). Ultimately, this process promotes the transcription and expression of type 1 interferon (IFN) and nuclear factor kappa B (NF-κB) (**Figure 1**) (13). It is noteworthy that cGAS can also recognize host DNAs, including mitochondrial DNA (mtDNA) and nuclear DNA (nuDNA), which contribute to metabolic dysfunction and sterile inflammation (14, 15).

The cGAS-STING pathway, initially extensively researched in the context of pathogenic infections (16–19), has been identified as a significant factor in various ischemic diseases (20, 21). By around 2020, its importance in ischemic stroke started to gain broader acknowledgment in the scientific literature (22). This recognition has been bolstered by a growing body of evidence highlighting the cGAS-STING pathway’s crucial involvement in neuroinflammation, a primary contributor to cerebral ischemic injury (23–27).

Research on immunotherapy as an intervention for cerebral ischemic injury has gained significant momentum (28, 29). Nevertheless, the blood-brain barrier (BBB) and the lack of cerebral lymphatic vessels significantly hinder the innate immune system’s function in the central nervous system (CNS), leading to complex interactions between the CNS and the immune system in the context of ischemic stroke (30). The cGAS enzyme is primarily distributed in microglia within the brain, directly contributing to the immune response in the CNS (31). Furthermore, metabolic dysfunction-induced cell injury has the potential to directly trigger the activation of the central and peripheral cGAS-STING pathway, instigating an inflammatory response in the corresponding regions (32–34), and increasing susceptibility to ischemic stroke. Additionally, the DNA structure of neutrophil extracellular traps (NETs) is widely thought to exacerbate the negative effects of thrombolysis by modulating the cGAS-STING pathway (24, 35). These findings offer insights into the intricate molecular mechanisms underlying immune cell-to-cell interactions. Accordingly, the cGAS-STING pathway represents a promising avenue for further exploration in immunotherapy for ischemic stroke.

This comprehensive review aims to provide an overview of the importance of the cGAS-STING pathway in ischemic stroke, emphasizing the neuroinflammatory responses resulting from its over-activation. While numerous comprehensive reviews published over the past few years have thoroughly examined the mechanisms of the cGAS-STING pathway, the present review provides a comprehensive analysis of the intricate mechanisms through which the cGAS-STING pathway contributes to the heightened susceptibility and detrimental effects of ischemic stroke. It also presents a novel perspective on its involvement in regulated cell death (RCD), acknowledging that the precise underlying mechanisms remain to be fully elucidated. To the best of our knowledge, this is the most comprehensive review about the GAS-STING pathway in ischemic stroke, encompassing risk factors, pathophysiology, potential therapeutic targets, and the side effect of thrombolytic therapy.

## 2 The overview of cGAS-STING pathway

In the context of the innate immune system, a range of intracellular and extracellular danger signals (36) are recognized by various PRRs. These PRRs include transmembrane Toll-like receptors (TLRs), NOD-like receptors (NLRs), cytoplasmic RIG-I-like helicases (RLHs), and cGAS (37, 38). Several PRRs are capable of detecting and responding to DNA, including Toll-like receptor 9 (TLR9), Absent in Melanoma 2 (AIM2), Z-DNA binding protein 1 (ZBP1), Interferon gamma-inducible protein 16 (IFI16), and cGAS (39–43). Typically, host DNA is confined to the nucleus or mitochondria. Furthermore, cells are capable to identify and remove superfluous DNA. This is achieved through the action of various enzymes such as DNase I, located in the extracellular space, DNase II, located in the nucleus, and three prime repair exonuclease 1 (TREX1, originally designated DNase III), located in the cytoplasm. These enzymes play a crucial role in regulating the quantity and distribution of cellular DNA (44). However, instances such as genomic DNA instability, mitochondrial stress, and lysosomal rupture can lead to the discharge of DNA into the cytoplasm (45–47). The activation of DNA sensors and downstream pathways can occur due to a reduction in endogenous DNase activity, an escalation in DNA damage, or an upsurge in exogenous DNA (48).

cGAS, the DNA sensor, a 60kDa protein, exhibits a high degree of sensitivity towards dsDNA, and its ability to recognize DNA is sequence-independent, allowing for indiscriminately binding and sensing the dsDNA (49). The cGAS protein comprises three domains, namely a DNA binding domain located at its N-terminus, a central nucleotidy transferase (NTase) domain, and a C-terminal domain (50). The interaction between the C-terminus of cGAS and the phosphate backbone of dsDNA is facilitated by a conserved zinc ribbon (10, 51). Upon binding to dsDNA, the resulting 2:2 complex of cGAS and DNA undergoes conformational changes that are believed to be catalytic in nature. Notably, cGAS dimers are situated between two distinct dsDNA fragments, thereby forming a stable complex (49). In contrast, single-stranded DNA and double-stranded RNA are incapable of forming a stable complex, thus confirming that the specificity of cGAS for dsDNA is determined by the structure of this stable complex (49). In addition to dsDNA, cGAS can also be activated by long-interspersed element-1 (LINE-1) and high-mobility group box 1 (HMGB1), influencing its downstream pathways (52, 53). Further upstream activators of cGAS remain to be identified in future studies. It is noteworthy that certain viruses, including vesicular stomatitis virus and herpes simplex virus do not directly elicit the activation of cGAS via their DNA or RNA. Rather, these viruses can indirectly stimulate cGAS by releasing mtDNA (54).

The localization of cGAS extends beyond the cytoplasm to include the cell and nuclear membranes (55). Within the membrane, cGAS exhibits heightened proficiency in detecting exogenous DNA. In the cytoplasm, the release of mtDNA resulting from Bak or Bax-mediated mitochondrial damage, coupled with the upregulation of mitochondrial transcription factor A (TFAM), enhances cGAS sensitivity to long DNA, ultimately leading to cGAS activation (53, 56). In the context of senescent cells, cGAS is observed to infiltrate chromatin as a result of micronucleus instability or nuclear membrane rupture, thereby instigating the activation of pro-inflammatory genes (14). The cGAS-STING pathway is closely associated with cellular senescence, predominantly by promoting the induction of senescence (14). This protective mechanism, which serves as a safeguard against the development or reception of tumor cells, emphasizes the significance of the cGAS-STING pathway ‘s distinctive function in modulating senescent cell activity. Moreover, cGAS is also identified within the cellular nucleus, predominantly in an inactive or low-active state under physiological conditions (57). Recent findings underscore that a mechanism exists within the nucleus that inhibits cGAS activity. This inhibition is attributed to the barrier-to-autointegration factor 1 (BAF) that dynamically competes with cGAS for DNA binding, effectively obstructing the activation complex formation between DNA and cGAS (58). Such a mechanism serves as a protective strategy, ensuring precision in cGAS’s discrimination of genomic DNA.

Upon formation of the cGAS-dsDNA complex, ATP and GTP are recruited to cyclize and create two phosphodiester bonds, ultimately yielding a distinctive 2′3′-cGAMP structure is crucial for STING activation (59). STING, a 42 KD protein with four transmembrane domains, predominantly localizes as a dimer on the ER (60). Upon binding of cGAMP, a conformational change occurs in the C-terminal structural domain of STING (11), which triggers its translocation from the ER to the Golgi apparatus. This process necessitates the assembly of Coat Protein Complex II (COPII) vesicles, involving several proteins, including Sar1, Sec13, Sec24, and Sec31 (61–64). The regulation of downstream pro-inflammatory cytokines is contingent upon the relocation of STING to the Golgi. Upon STING translocation to the Golgi apparatus, activates the TBK1 and IRF3. STING’s oligomerization and activation of TBK1 are facilitated by its palmitoylation (65). Subsequently, IRF3 was phosphorylated and dimerized by TBK1, which then translocates to the cell nucleus to induce IFN-1 expression (65). Then, IFN-1is recognized by cell membrane receptor IFNAR, composed of IFNAR1 and IFNAR2 subunits, leading to the activation of the JAK/STAT pathway (66). This results in the formation of specific complexes that regulates a group of target genes containing the interferon-stimulated response element (ISRE), thereby regulating the expression of interferon-stimulated gene factors (ISG) (**Figure 1**) (67). Furthermore, STING that has undergone maturation is subjected to lysosomal degradation via autophagy, indicating an interaction between the cGAS-STING pathway and autophagy (61). The cGAS-STING pathway not only mediates the expression of interferons but also activates inhibitors of kappa B kinase (IKK) via STING, resulting in the production of NF-κB (68). Nevertheless, the regulatory mechanisms of non-IRF3 transcription factors remain elusive.

## 3 Role of cGAS-STING pathway in the risk factors of ischemic stroke

Prolonged and persistent inflammation poses a heightened risk of stroke, especially in individuals with coexisting medical conditions such as hypertension, hyperglycemia, and hyperlipidemia. Mitigating modifiable risk factors is imperative in the prevention, management, and recurrence of ischemic stroke. Recent studies have identified that these metabolic aberrations are often accompanied by the dysregulated activation of the cGAS-STING pathway in various tissue cells, especially in endothelial cells (69, 70). Mitochondrial dysfunctions, especially those leading to the aberrant release of mtDNA, are prominent indicators of metabolic stress (71). Such mitochondrial disturbances can give rise to mtDNA damage and abnormal distribution, thereby suggesting a potential association between mitochondrial-originated metabolic dysfunction and the cGAS-STING pathway, as evidenced by various studies (15, 32, 72). In parallel, senescent cells, both under physiological and pathological scenarios, might augment DNA exposure due to their compromised membrane integrity and diminished genomic DNA repair capabilities (14, 73), thus influencing the cGAS-STING pathway’s activation. This section underscores the importance of the cGAS-STING pathway-mediated chronic inflammatory response in the risk factors linked to ischemic stroke.

### 3.1 Hypertension

Hypertension, which accounts for 52% of all cases, is the most significant independent risk factor for ischemic stroke and is therefore considered a crucial target for long-term prevention of stroke (74). The American Stroke Association recommends anti-hypertensive therapy for patients eligible for intravenous (IV) thrombolysis with recombinant t-PA. The pre-treatment systolic BP should not exceed 185 mmHg, and the diastolic BP should not exceed 110 mmHg. Furthermore, it is recommended to maintain BP levels below 180/105 mmHg during the first 24 hours following treatment (75).

The renin-angiotensin system (RAS) is a fundamental molecular mechanism that initiates hypertension, and its hyperactivation is facilitated by sympathetic nerves (76). Angiotensin II (Ang II), a crucial constituent of the RAS, is produced in multiple tissues, including the circulation, blood vessels, kidneys, and brain tissues. In the brain, Ang II stimulates microglia polarization towards the M1 state and induces the release of various pro-inflammatory cytokines (77). Research has indicated that the release of these cytokines is closely linked to an increased number of damaged mitochondria and intracytoplasmic dsDNA (78). Activation of the cGAS-STING pathway, predominantly situated in the paraventricular nucleus and rostral ventrolateral medulla of the brain, governs mitophagy and pro-inflammatory cytokines release, thereby inducing microglial polarization and localized neuroinflammation (33, 78). The ensuing neuroinflammation in the central cardiovascular system elicits sympathetic excitation, which ultimately contributes to the long-term development of hypertension. The results of utilizing an intracisternal infusion of RU.521, an inhibitor of cGAS, indicated a reduction in microglia polarization, a decrease in neuroinflammation, a lowered sympathetic excitability, and a significant decrease in BP (33). These findings suggest that the cGAS-STING pathway regulates BP by promoting neuroinflammation in the central cardiovascular system.

Activation of the cGAS-STING pathway occurs not only in the cardiovascular center but also in the peripheral tissues of hypertensive patients, thereby exacerbating tissue damage. Research has demonstrated that direct inhibition of cGAS can enhance left ventricular contractility and alleviate ventricular hypertrophy in pressure-overload-induced heart failure (79). These findings indicate that the controlled expression of cGAS has a direct impact on myocardial contractile function and cardiac remodeling, influencing BP and delaying the onset of heart failure. Moreover, augmented STING expression has been detected in cardiomyocytes and is closely associated with heart failure (79, 80). Ventricular cells have been shown to be influenced by Ang II in terms of cGAS activation and STING upregulation, and the inhibition of ER stress has been found to substantially decrease cGAS-STING pathway activation (81). These suggest that the cGAS-STING pathway is directly involved in the development of Ang II-induced hypertension, which ultimately results in damage to peripheral tissues.

To date, several mechanisms by which the cGAS-STING pathway influences BP have been identified, such as direct involvement in the regulation of cardiovascular centers, directly affecting myocardial contractile function, and cardiac remodeling. Further exploration of the cGAS-STING regulatory pathway may offer new insights into blood pressure modulation. Additionally, whether the activation of the cGAS-STING pathway is closely associated with tissue damage beyond the myocardium in the context of hypertension remains to be determined.

### 3.2 Hyperglycemia

Hyperglycemia represents an additional modifiable and independent risk factor for stroke. A J-shaped association between glycosylated hemoglobin levels and the likelihood of stroke has been documented (82). Hyperglycemia frequently co-occurs with vascular remodeling, and the architecture of the microvasculature influences the development of collateral circulation during the onset of ischemic stroke, which in turn tends to augment the size of the infarct (83). Furthermore, blood glucose levels exhibit a positive correlation with anaerobic metabolism, as heightened blood glucose levels lead to an accumulation of lactic acid and acidosis during ischemia (84).

The cGAS-STING pathway has been implicated in the regulation of blood glucose. Notably, the expression of cGAS, STING, and TBK1 is elevated in adipocytes, which may contribute to sterile inflammatory response, insulin resistance, and hyperglycemia (32). Furthermore, a high-fat diet (HFD) leads to the release of mtDNA, which activates the cGAS-STING pathway and promotes chronic inflammation (85). In the islet, activation of the cGAS-STING pathway in β-cells of HFD mice has been found to inhibit glucose metabolism and increase the load on islet β-cells (86). Following intervention with the STING inhibitor C176, the inflammatory response was significantly downregulated, thereby safeguarding the proper functioning of pancreatic β-cells and glucose metabolism. Disulfide-bond A oxidoreductase-like protein (DsbA-L), an enzyme that exhibits an inverse correlation with obesity in both mice and humans (87), has been found to impede the entry of mtDNA into the cytoplasm, suppress the activation of the cGAS-STING pathway, and mitigate the inflammatory response of adipose tissue under HFD conditions (15). The downregulation of STING has been demonstrated to decrease adipose tissue inflammation and insulin resistance induced by an HFD (88). The modulation of the cGAS-STING pathway as a means of regulating insulin resistance presents a novel approach to the treatment of type 2 diabetes. Additionally, the activation of the cGAS-STING pathway has been observed in the microglia of HFD mice, concomitant with microglia polarization and neuronal damage (89). This suggests that the cGAS-STING pathway may serve as a novel “bridge” linking metabolic dysfunction and neuroinflammation. Moreover, existing evidence has demonstrated that hyperglycemia has an impact on the production of vascular endothelial cells, potentially through the activation of the cGAS-STING pathway and the dysregulation of the Hippo-Yes-associated protein (YAP) pathway (90). Mammalian Ste20-like kinases 1 (MST1) have been identified as a contributing factor to YAP phosphorylation, which inhibits angiogenesis. Activation of the cGAS-STING pathway by mtDNA leads to the production of IRF3, which acts on the promoter region of MST1 to induce its expression and ultimately inhibit angiogenesis (90). To summarize, the excessive activation of the cGAS-STING pathway is linked to hyperglycemia-induced chronic inflammation, metabolic abnormalities, and impaired angiogenesis, resulting in inflammation and compromised blood flow in the brain.

### 3.3 Hyperlipidemia and atherosclerosis

Hyperlipidemia, a well-established risk factor for ischemic stroke, is a significant contributor to the development of atherosclerosis (AS). It is characterized by a subacute immune-mediated inflammatory response resulting from the accumulation of lipids around the vessel wall, which triggers macrophages to migrate and engulf the lipids, ultimately leading to the formation of atherosclerotic plaques (91). Furthermore, the endothelium serves as the primary defense against AS, and the process of endothelial-to-mesenchymal transition (EndMT) is closely associated with the pathogenesis of AS (92). Hence, the crucial intervention strategy for ischemic stroke involves mitigating hyperlipidemia-induced endothelial cell dysfunction, which leads to anomalous cerebral blood flow.

**Figure 2:**
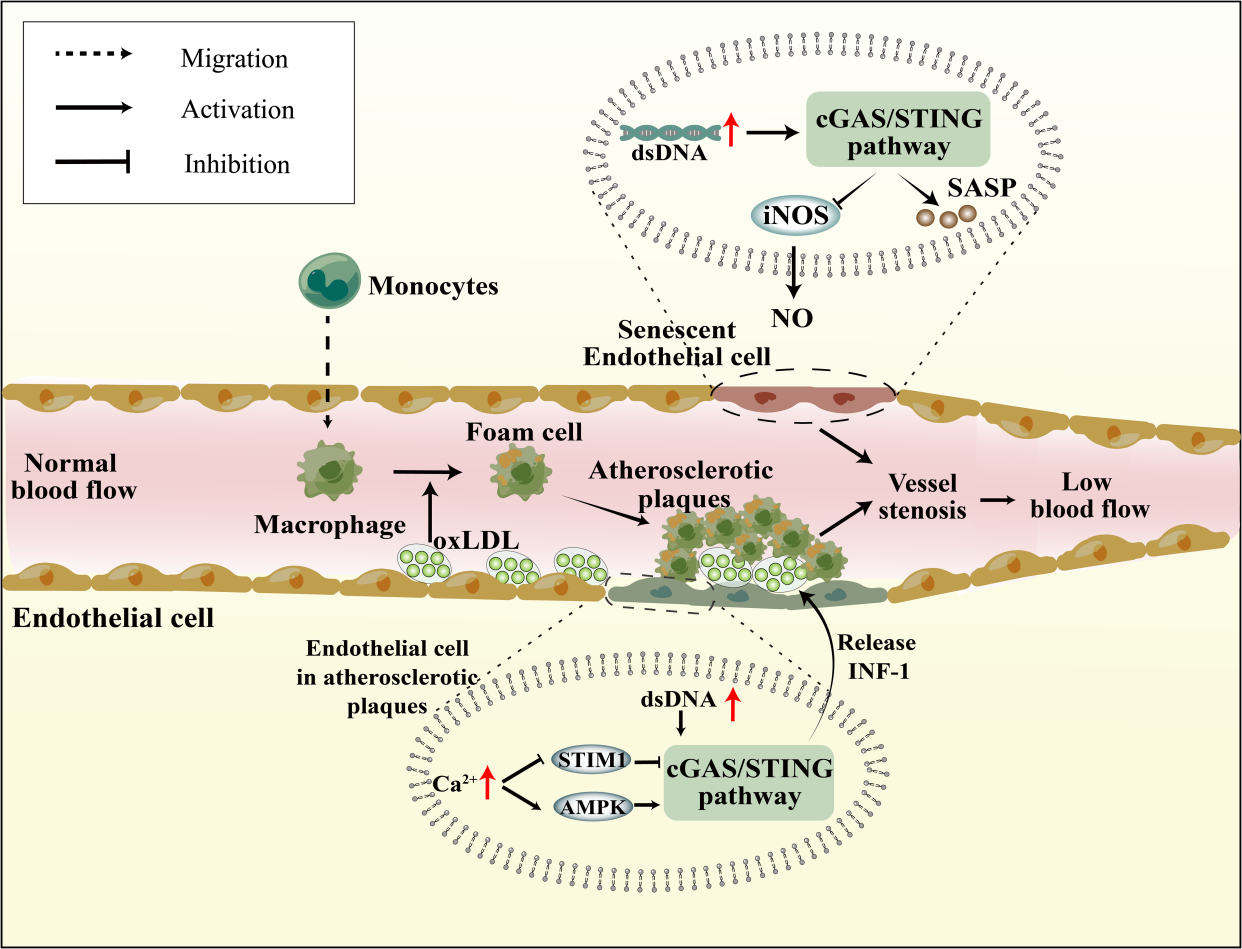
Senescence and atherosclerosis affect vascular endothelial function through cGAS-STING pathway. In Senescence cells, mitochondrial dysfunction, reduced nuDNA stability, and impaired DNA damage repair mechanisms lead to intracellular DNA accumulation, subsequently activating the cGAS-STING pathway. This not only results in the reduction of iNOS and NO, affecting vasodilation, but also promotes SASP production, impacting endothelial cell function. Atherosclerotic plaques lead to endothelial cell damage, elevated Ca^2+^ levels inhibit the suppressive role of STIM1 on the cGAS-STING pathway and also promote AMPK-mediated activation of the cGAS-STING pathway. Furthermore, mtDNA resulting from mitochondrial damage also activates the cGAS-STING pathway.

In the context of vascular inflammation research, it was noted that AS plaques exhibited a high expression of STING (93). Oxidized low-density lipoprotein (oxLDL) accumulates in the vasculature, leading to the recruitment of circulating immune cells, subsequently triggering an inflammatory cascade that culminates in the formation of atherosclerotic plaques (94) (**Figure 2**). Recent studies have identified mislocalized self-DNA and DNA damage in AS (95, 96), with this DNA predominantly localized within AS plaques (97). The activation of the cGAS-STING pathway was observed in dysfunctional endothelial cells with elevated cytosolic mtDNA and nuDNA, leading to an increase in the expression of IL-6 mRNA in AS (98). Moreover, research indicates that damaged endothelial cells within these plaques, due to mitochondrial dysfunction, exhibit significantly elevated mtDNA levels, inducing the activation of the cGAS-STING pathway, thereby exacerbating endothelial cell damage (99). The functionality of endothelial cells is largely contingent upon Ca^2+^ concentrations. Stromal interaction molecule 1 (STIM1), an essential regulator of calcium homeostasis, orchestrates the equilibrium of Ca^2+^ levels between the cytoplasm and ER (100). Under physiological conditions, STIM1 supports the tethering of STING to the ER, suppressing the subsequent activation of the cGAS-STING pathway and attenuating the type 1 IFN response (101). Hyperglycemia induces elevated Ca^2+^ concentrations in endothelial cells (102). Similarly, in the context of AS, calcium homeostasis is compromised, leading to the displacement of STIM1 and diminished STING inhibition (103). The apolipoprotein E (ApoE) -/- mice subjected to HFD, which induces the formation of atherosclerotic plaques, it also observed an elevation in Ca^2+^ levels. This in turn activates calcium/calmodulin-dependent protein kinase II (CaMKII), leading to the activation of AMP-activated protein kinase (AMPK) and subsequent stimulation of the STING pathway (**Figure 2**) (104). In addition, activation of STING in macrophages also promotes AS. In HFD ApoE-/- mice, attenuation of atherosclerotic lesions was observed upon knocking down STING or reducing macrophage accumulation (105). Moreover, the administration of the STING-specific antagonist C-167 to ApoE-/- mice led to a decrease in plaque lesions and lipid accumulation (106). Furthermore, the process of EndMT results in the impairment of endothelial cells, thereby facilitating the development of atherosclerotic plaque. Upon induction of EndMT in human aortic endothelial cells by palmitic acid (PA), activation of the cGAS-STING pathway was observed, and the suppression of cGAS expression mitigated inflammation, oxidative stress, and EndMT (107). Modulating the activation of the cGAS-STING pathway in endothelial cells may represent a novel molecular mechanism to ameliorate atherosclerotic injury.

### 3.4 Senescence

The incidence of ischemic stroke and associated mortality exhibit a marked rise with advancing age (108, 109). Vascular endothelial dysfunction and neuronal degeneration are the primary pathophysiological mechanisms underlying stroke. Recent studies have shed light on the neurodegenerative damage mediated by the cGAS-STING pathway in the context of neural aging (110–112). In this regard, our research endeavors to investigate the involvement of the cGAS-STING pathway in senescent vasculature.

The expression levels of cGAS, STING, and p-IRF3/IRF3 in endothelial cells exhibit an increase with age, while the expression of inducible nitric oxide synthase (iNOS) and nitric oxide (NO) production, a significant vasodilatory factor, decrease in an age-dependent manner (70). The inhibition of the cGAS-STING pathway has been observed to mitigate the disparity in eNOS and NO expression between 12-month and 6-month-old mice, indicating that the modulation of the cGAS-STING pathway can directly enhance senescent vascular structures, cerebral blood supply, and reduce nervous system injury (**Figure 2B**) (70). Senescent cells demonstrate distinct molecular and functional alterations, including decreased stability of nuDNA, mitochondrial dysfunction, aberrant generation of reactive oxygen species (ROS), and compromised DNA damage repair mechanisms (113). A recent publication in *Nature* regarding senescence underscored the impact of YAP/TAZ activity and the activation of the cGAS-STING pathway on cellular senescence (114). The involvement of YAP/TAZ in the transcriptional regulation of lamin B1 and ACTR2, which respectively contribute to the structural integrity of the nuclear membrane and the formation of the peri-nuclear actin cap (115, 116), implies the importance of YAP/TAZ in the modulation of cellular senescence. Specifically, YAP/TAZ may exert its effect by preserving the integrity of the nuclear membrane, thereby indirectly suppressing the activation of the cGAS-STING pathway.

The senescence-associated secretory phenotype (SASP) is a crucial aging marker that can modify intercellular communication levels, thereby inducing senescent cells and their neighboring cells to age more rapidly, resulting in chronic low-grade inflammation. Senescent endothelial and smooth muscle cells exhibit an increase in SASP expression, which is accompanied by abnormalities in endothelial secretory function and vascular architecture (117). Moreover, the cGAS-STING pathway recognizes DNA fragments and facilitates the release of SASP from senescent cells (**Figure 2B**) (14, 118). The inhibition of cGAS, STING, or NF-κB has been demonstrated to eliminate SASP gene expression in both mice and human cells (119). Additionally, a novel cellular senescence phenotype, characterized by the response of type 1 IFN, has been observed to maintain high SASP expression in senescent cells. It may be linked to the elevated expression of LINE-1 in senescent cells, which possesses high reverse transcriptase activity and transcribes mRNA into DNA in the cytosol (120). Despite the identification of a positive correlation between the cGAS-STING pathway and SASP, the precise molecular mechanism underlying this relationship remains unknown.

## 4 Role of cGAS-STING pathway in pathophysiology of ischemic stroke

Ischemic stroke encompasses diverse pathophysiological mechanisms, including oxidative stress, ER stress, neuroinflammatory response, and RCD. The over-activation of the cGAS-STING pathway may represent a novel mechanism that exacerbates these processes. Given the non-regenerative nature of nerve cells and their susceptibility to ischemia and hypoxia, identifying strategies to inhibit the pathophysiological cascade of ischemic stroke and reverse neuronal death could have significant implications for limiting the expansion of the penumbra and treating ischemic stroke.

### 4.1 Oxidative stress

The brain is a high-energy-demanding organ that can only be met through oxidative phosphorylation. Both cerebral ischemia injury and cerebral ischemia/reperfusion injury (CI/RI) can induce oxidative stress, which generates ROS that directly damage DNA, lipids, and proteins, leading to cell death. Under normal conditions, DNase I, DNase II, and TREX1 can degrade a small amount of damaged DNA, rendering the DNA sensor inactive (121). However, oxidatively damaged DNA is more resistant to nuclease degradation, resulting in its accumulation in the cytoplasm (122, 123).

**Figure 3:**
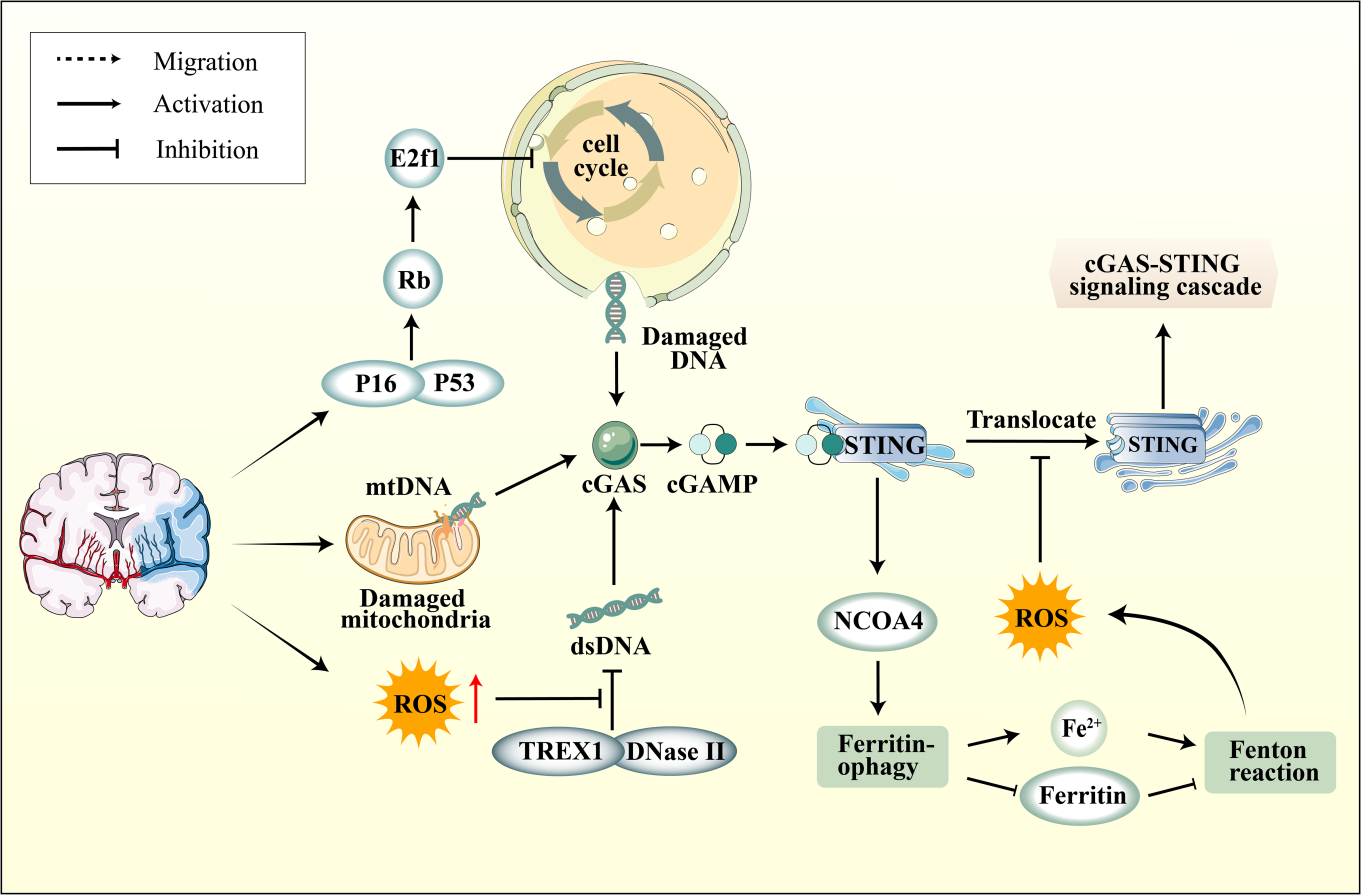
The connection between cGAS-STING pathway and oxidative stress. Ischemic stroke induces elevated expression of P16 and P53 in neurons within the ischemic region, subsequently modulating the cell cycle through the regulation of Rb and E2f1, leading to DNA damage. On another front, mitochondrial damage also results in increased cytoplasmic mtDNA levels. Furthermore, ROS enhances the resistance of DNA to degradation by nucleases, including DNase II and TREX1, leading to the accumulation of DNA in the cytoplasm. Subsequently, activated STING upregulates NOCA4 expression, leading to ferritinophagy and a surge in ROS production via the Fenton reaction. However, ROS can impede the phosphorylation of STING Cys147, thereby hindering the downstream formation of the protein complex involved in innate immune signaling by inhibiting the transfer of STING from the ER to the Golgi apparatus.

Recent studies have confirmed a complex relationship between oxidative stress in ischemic stroke and the cGAS-STING pathway. Initially, ischemic stroke leads to an excess of ROS within neurons, resulting in cell cycle aberrations, DNA damage, and increased nuclear DNA content. Ischemic stroke also elevates Rb levels, subsequently inducing dysfunction of the cell cycle-inhibitory transcription factor E2f1 (124). Moreover, ischemic stroke activates p38 MAPK, subsequently enhancing p53 and p16 expression in the infarct area, further modulating the cell cycle through Rb and E2f1 regulation (125–127). Cellular DNA damage caused by ischemic stroke is closely associated with in activating the cGAS-STING pathway. Additionally, as the primary site of oxidative respiration, mitochondrial damage during ischemic stroke also leads to increased cytoplasmic mtDNA, thereby activating cGAS (128). Current research reveals that dysregulation of iron metabolism is a crucial aspect of ischemic stroke and is closely linked to oxidative stress. In the early stages of I/R, Nuclear Receptor Coactivator 4 (NCOA4) mediated ferritinophagy results in elevated cytoplasmic Fe2+, initiating oxidative stress, which further promotes autophagy and apoptosis, culminating in augmented brain injury (129). An in-depth investigation into NCOA4 and the cGAS-STING pathway revealed that activating the cGAS-STING pathway can exacerbate ischemic brain damage via NCOA4-mediated ferritinophagy (130) (**Figure 3**).

In fact, NCOA4-mediated ferritinophagy ultimately induces the Fenton reaction, leading to elevated intracellular ROS levels. The ROS can oxidize the cysteine residue at position C147 of the STING protein, thereby inhibiting the cGAS-STING pathway and subsequently downregulating the expression of IFN-1 (131). The influence of ROS on the cGAS-STING pathway has been further highlighted within the realm of ferroptosis research. Ferroptosis is a distinct oxidative stress-driven cell death mechanism marked by the depletion of glutathione peroxidase 4 (GPX4), an essential protein that counteracts cellular ferroptosis. In the absence of GPX4, there is enhanced cellular lipid peroxidation, which subsequently suppresses the cGAS-STING pathway (132). The mechanism suggests that the upsurge of ROS levels and oxidative stress, which induces STING carbonylation at C88 and impedes its translocation from the ER to the Golgi complex, hindering the downstream signaling of the cGAS-STING pathway (132) (**Figure 3**). This suggests that oxidative stress can, on one hand, impact the cell cycle and mitochondrial function, subsequently activating the cGAS-STING pathway via DNA. On the other hand, ROS can also inhibit STING activation. The intricate relationship between the cGAS-STING pathway and oxidative stress is pivotal, and the interplay between the two requires further elucidation.

Crucially, the interplay between the cGAS-STING pathway and oxidative stress manifests divergent outcomes across various ischemic disorders. For example, the administration of the cGAS inhibitor RU.521 successfully mitigated oxidative stress-induced harm and apoptosis in lung schemia/reperfusion (I/R) injury by suppressing the cGAS-STING pathway (133). In contrast, the activation of cGAS was found to confer protection against ischemic liver injury by initiating autophagy, which effectively inhibited oxidative stress (134). The bidirectional regulation of oxidative stress by the cGAS-STING pathway suggests a unique characteristic that has significant implications for innate immunity, enhancing its defensive efficacy against oxidative stress in ischemic stroke. To comprehensively comprehend the pathway’s involvement in diverse biological processes, further investigation is warranted to identify the cellular substructures implicated.

### 4.2 ER stress

The induction of ER stress is a consequential mechanism that leads to neuronal cell damage. During cerebral ischemia and hypoxia, changes occur within the ER, resulting in the abnormal aggregation of unfolded proteins and triggering the unfolded protein response (UPR). The accumulation of unfolded proteins prompts the activation of PKR-like ER-resident kinase (PERK), activating transcription factor-6 (ATF6) and inositol-requiring enzyme 1α (IRE1α), which bind to these proteins and undergo phosphorylation, thereby initiating downstream pathways that culminate in apoptosis (135).

The interplay between the cGAS-STING pathway and ER stress has been well-documented. In studies on pathogen infections, the cyclic-di-adenosine monophosphate found in live Gram-positive bacteria is known to activate STING, which in turn mediates ER stress and subsequently influences cellular autophagy (136). Moreover, research has indicated that several pharmacologic ER stress inducers, alongside oxygen-glucose deprivation, trigger the activation of IRF3. Notably, the application of 4-(2-Aminoethyl)-Benzenesulfonyl Fluoride Hydrochloride (an inhibitor targeting the unfolded protein ATF6) markedly curtails IRF3 activation (137). This research suggests that ischemic disorders might activate the cGAS-STING pathway through ER stress or the UPR (137). Parallel findings are reported in various ischemic conditions. In studies concerning lung I/Rinjury, it was observed that cGAS-STING pathway expression is upregulated in alveolar epithelial type II cells of I/R rats. When the cGAS-STING pathway was activated, ER stress was induced in alveolar epithelial type II cells, whereas inhibiting the cGAS-STING pathway prominently ameliorated both ER stress and I/R injury (133). Additionally, mice manifesting cardiac hypertrophy displayed augmented markers of PERK and eIF2α ER stress, markers which were significantly attenuated upon STING knockout (81). Subsequent studies established that in neurons, ER stress could instigate the cGAS-STING pathway, culminating in the polarization and activation of microglia (138). The use of the PERK inhibitor GSK2656157 substantially reduced the production of IFN and the polarization of microglia towards the M1 phenotype (138). Intriguingly, STING gain-of-function mutations were associated with anomalous expression of multiple unfolded proteins, including Chop and p-IRE1 (139). In the context of ischemic stroke, a similar accumulation of these unfolded proteins is observed. We posit that the aberrant accumulation of unfolded proteins in ischemic stroke may likely be attributed to the dysregulation of the cGAS-STING pathway. Modulating the activation of this pathway might be pivotal in intervening the ER stress observed in ischemic stroke.

### 4.3 Neuroinflammation

#### 4.3.1 Aberrant cGAS-STING pathway exacerbates neuroinflammation in ischemic stroke

Neuroinflammation is an inflammatory response that manifests in the brain or spinal cord and is observed in various cerebrovascular diseases, particularly ischemic stroke, leading to the disruption of immune homeostasis and aggravation of organ damage (140). Neuroinflammation plays a dual role in ischemic stroke, as it can both amplify ischemic damage and contribute to infarct resolution (141). Microglia activation is widely acknowledged as a crucial event in neuroinflammation, and the high expression of cGAS in microglia has been verified (24). Hence, regulating the activation of the cGAS-STING pathway in microglia is critical in regulating neuroinflammatory responses during ischemic stroke.

After an ischemic stroke, microglia, the immune cells residing in the brain, are activated and subsequently release inflammatory cytokines and chemokines. After the onset of an ischemic stroke, the intrinsic negative feedback mechanism between IFN-1 and interferon-stimulated genes ISGs in the CNS becomes dysregulated (142). Furthermore, IFN-1 induces the release of cytokines and chemokines, including chemokines (C-C motif) ligand-2 (CCL-2) and CCL-3, from activated microglia (143). The chemokines facilitate the clustering of peripheral macrophages and neutrophils at the injury site. In the event of cerebral ischemia, astrocytes, which is associated with maintaining normal brain function, initiate autophagy via the HIF-1/mTOR pathway (144), leading to endothelial cell dysfunction and astrocyte autophagy, which in turn increases BBB permeability, thereby promoting the infiltration of peripheral immune cells into the brain. In summary, microglia are pivotal in the neuroinflammatory response to ischemic stroke.

It is now understood that the cGAS-STING pathway can prompt polarization of microglia/macrophages towards the proinflammatory M1-like phenotype. Studies have revealed that cGAS governs macrophage polarization in repairing cardiac injury in myocardial infarction (21). The absence of cGAS function has been demonstrated to encourage the transformation of macrophages to a reparative phenotype, which restrains pathological remodeling and advances angiogenesis and early survival (21). In fact, microglia, which are highly specialized macrophages, also have their M1/M2 polarization modulated by the cGAS-STING pathway.

The cGAS-STING pathway exacerbates cerebral ischemic injury has been confirmed through experiments using si-cGAS inhibition of microglial M1 polarization in a middle cerebral artery occlusion (MCAO) mouse model, resulting in the downregulation of the M1/M2 ratio and attenuation of neuroinflammation in ischemic stroke by suppressing the shift in microglia phenotype (23). A similar cGAS-mediated neuroinflammatory response was observed in CI/RI (145). The activation of the microglial cGAS-STING pathway in the ischemic zone has been proposed to contribute to the local microenvironment. The upregulation of cGAS expression in microglia by HDAC3 and the facilitation of downstream P65 acetylation and nuclear localization resulted in increased pro-inflammatory cytokines (145). Furthermore, within the context of subarachnoid hemorrhage, a specific type of stroke, a significant increase in STING expression was observed 12 hours post-injury (146). The administration of CMA, a STING agonist, resulted in heightened neuronal damage, whereas using C-176, a STING inhibitor, produced neuroprotective outcomes, such as diminished brain edema, mitigated neuronal damage, and reduced microglial activation (146). Additionally, STING expression was upregulated in a neonatal hypoxic-ischemic encephalopathy rat model, and the implementation of STING inhibitors yielded a significant reduction in infarct size and neuroinflammation (52).

These findings suggest that targeting the cGAS-STING pathway may be a viable immunotherapeutic approach for treating ischemic stroke. Specifically, the polarization of microglia mediated by cGAS-STING may represent a novel mechanism for inducing neuroinflammation. Additionally, activated microglia can directly release pro-inflammatory cytokines and indirectly attract immune cells from peripheral regions toward the ischemic zone, thereby promoting neuroinflammation. Consequently, targeting the cGAS-STING pathway may offer a promising avenue for immunotherapy in treating ischemic stroke.

#### 4.3.2 Therapeutical potential of targeting cGAS-STING pathway against neuroinflammation in ischemic stroke

It is widely acknowledged that neuroinflammation involves the activation of microglia and astrocytes and the production of pro-inflammatory cytokines that trigger nerve cell damage through various signaling pathways and exacerbate ischemic stroke (147). There are preclinical compounds that can potentially mitigate ischemic stroke by modulating inflammatory response pathways or targets (148–150). Presently, there are surge in research on the involvement of the cGAS-STING pathway mediating neuroinflammation in the context of ischemic stroke. These findings have prompted research into the potential therapeutic applications of this innate immune pathway in treating ischemic stroke. Targeting the cGAS-STING pathway represents a promising novel approach to immunotherapy for neuroinflammatory disorders. Although several pharmacological studies have now confirmed that DNA inhibitors can intervene in neuroinflammation, such as A151 and DNase I (22, 24, 25). However, it remains to be further determined whether other inflammatory pathways are also affected as the cGAS-STING pathway.

The regulation of cGAS expression in microglia has the potential to affect the activation of microglia and the downstream pro-inflammatory cytokines, consequently influencing the neuroinflammatory response in ischemic stroke. HDAC3 transcriptionally promoted the expression of cGAS and potentiated the activation of the cGAS-STING pathway by regulating the acetylation and nuclear localization of p65 in microglia (145). Additionally, the induction of mitochondrial damage by ischemia-reperfusion further increased mtDNA content in microglia, leading to enhanced microglia activation via the cGAS-STING pathway. In the MCAO model, the administration of HDAC3 inhibitors TSA/MS275/RgFP109/RgFP966 effectively mitigated the neuroinflammatory response by inhibiting the HDAC3-P65-cGAS axis (145). In the context of ischemic stroke, the DNA backbone of NETs has the ability to function as a ligand, thereby activating cGAS and subsequently inducing microglia activation and neuroinflammation (24). The administration of DNase I has been observed to mitigate cGAS activation and confer neuroprotective effects (24). Furthermore, A151 functions as a potent inhibitor of cGAS, effectively impeding its activation by competitively binding to DNA. A151 inhibits neuroinflammatory responses by inhibiting the production of inflammasome AIM2, consequently reducing neuronal cell death in ischemic stroke (22). The presence of the BBB poses challenges in effectively delivering drugs to the brain for therapeutic purposes. To address this issue, a novel engineering approach involving the integration of a CXCL12 biomimetic decoy into a versatile immunosuppressive nanoparticle, loaded with A151, has been developed (25). This nanoparticle specifically targets cGAS activity in microglia and hinders the infiltration of peripheral neutrophils and mononuclear macrophages. In an experimental rat model of ischemic stroke, the administration of A151 via the nanoparticle significantly suppressed the expression of Caspas-1 and modulated microglia polarization, resulting in a reduction in both infarct volume and neuronal damage (25). 4-sulfonic calixarenes target multiple dsDNA PRRs, including AIM2, cGAS and TLR9. In CI/RI model, 4-sulfonic calix[6]arene may be effective at both the dsDNA-binding site on cGAS and the 2′,3′-cGAMP, but not the CMA binding site on STING (151). RU.521, another inhibitor of cGAS, can increase the stacking interaction of cGAS amino-acid residues Arg364 and Tyr421 (152). Cerebral venous sinus thrombosis (CVST) is a potentially fatal cause of stroke. In a mouse model of CVST, the cGAS–STING pathway was upregulated and related to microglia pyroptosis and neuroinflammatory responses (27). Blocking cGAS with RU.521 suppressed the inflammatory cascade and NLRP3 inflammasome-mediated microglia pyroptosis, thereby exerting an anti-CVST effect and mitigating the occurrence of ischemic stroke (27). In the rat model of CI/RI, along with the inhibition of the cGAS by RU.521, the ferritinophagy, oxidative stress, autophagy, and apoptosis were inhibited, and CI/RI was ameliorated, which was attenuated by NCOA4 overexpression (130). In neonatal hypoxia-ischemia in a 10-day-old rat model, RU.521 can reduce the expression of cathepsin B and decrease the expression of Bax and caspase 3 cleavage (52).

C-176, the inhibitor of STING, also ameliorates the neuroinflammatory responses. C-176 blocks the STING palmitoylation by cysteine residue 91 (153). In both the *in vivo* MCAO rat model and the *in vitro* BV2 microglia oxygen-glucose deprivation/reperfusion model, C-176 effectively suppresses the polarization of microglia towards the M1 phenotype and the expression of downstream transcription factors, such as IRF3 and NF-κB (154). Nevertheless, studies in the stroke subtype, subarachnoid hemorrhage, have demonstrated that inhibiting the AMPK signaling can reverse the tissue and cellular ischemic injuries mediated by C-176, a specific STING agonist. Upon activation, STING phosphorylates and activates TBK1, which in turn inhibits AMPK activity. This leads to the activation of microglia, polarizing them into the M1 phenotype and resulting in the secretion of pro-inflammatory mediators and neuronal damage (146). It is widely accepted that N6-Methyladenosine (m6A) has been implicated in CI/RI (155). The expression of the microglial m6A demethylase fat mass and obesity-associated protein (FTO) was found to be significantly decreased in cerebral I/R injury. Conversely, the overexpression of FTO was observed to mitigate the inflammatory response mediated by microglia and reduce brain injury. This effect is believed to be achieved through the inhibition of cGAS expression, which is accomplished by reducing its mRNA stability via m6A modification (156). Activin A, a member of the transforming growth factor b (TGF-b) superfamily of growth and differentiation factors, promotes and maintains the survival of cortical neurons and protects neurons from neurotoxicity (157). In a mouse model of ischemic stroke, Activin A phosphorylates PKB via the PI3K-PKB pathway, which in turn PKB phosphorylates the S291 or S305 residue of mouse or human cGAS (158). This suggests that Activin A holds promise as a potential therapeutic agent for ischemic stroke by mitigating cGAS-STING-dependent neuronal autophagy. MicroRNAs (miRNAs) are ubiquitous 21-23nt RNA molecules in eukaryotes that repress mRNA translation by binding to the 3’-untranslated region (3’-UTR) of their target mRNAs (159). Studies have indicated that miR-340-5p exerts neuroprotective effects in SAH (a subtype of stroke) by modulating the expression of STING in microglial cells (160) (**Table 1**).

**Table 1:** Therapeutical Potential of Targeting the cGAS-STING Pathway against Neuroinflammation in Ischemic Stroke.

Target (s)	Intervention(s)	Mechanism	Subjects	References
HDAC3	TSA/MS275/RgFP109/RgFP966	Reduce neuroinflammatory responses by inhibiting the HDAC3-P65-cGAS axis	BV2 cells, HEK293T cells, and cGAS KO mice	(145)
NETs	DNase I	DNase I disrupts the DNA backbone of NETs, inhibiting the activation of the cGAS-STING pathway and microglia	HBMECs, Neutrophils, and cGas^−/−^ mice	(24)
cGAS	A151	A151 competes with DNA and inhibits the activation of cGAS, reducing the AIM2 inflammasome-mediated pyroptosis	BV2 cells and cGAS^f/+^ mice	(22)
	A151 by nanoparticle	A151 competes with DNA and inhibits the activation of cGAS, inhibiting the the expression of caspase-1 and microglia polarization	PC12 cells and C57bL/6 mice	(25)
	4-sulfonic calix[6]arene	4-sulfonic calix[6]arene affects at both the dsDNA-binding site on cGAS and the 2′,3′-cGAMP.	bone marrow-derived macrophages, Human monocyte-derived macrophages, and C57bL/6 mice	(151)
	RU.521	RU.521 increase the stacking interaction of cGAS amino-acid residues Arg364 and Tyr421, suppressing the pyroptosis-pertinent components, oxidative stress, and neurological deficits.	C57BL/6 mice	(27)
	RU.521	RU.521 block the cGAS inflammatory cascade, attenuating the NCOA4-mediated ferritinophagy.	C57BL/6 mice	(130)
	RU.521/LINE-1	RU.521 reducing the expression of cathepsin B and decreased the expression of Bax and caspase 3 cleavage. LINE-1 is a potential upstream activator of cGAS/STING pathway	Rat Model	(52)
	FTO	FTO inhibits cGAS expression through decreasing its mRNA stability via m6A modification, thereby alleviating inflammatory response in CI/RI	Mouse primary isolated microglia and male C57BL/6 mice	(156)
	Activin A	Activin A phosphorylates PKB via the PI3K-PKB pathway, which in turn PKB phosphorylates the S291 or S305 residue of mouse or human cGAS	Primary cortical neuron and C57BL/6 mice	(158)
STING	C-176	C-176 block the STING palmitoylation by cysteine residue 91, reducing the expression of IRF3 and NF-κB.	HT22 cells, BV2 cells, and C57BL/6 mice	(154)
	C-176	C-176 Inhibit the STING downstream and promote AMPK phosphorylation to attenuate microglia polarization and neuroinflammation	BV2 cells and C57BL/6 mice	(146)
	miR-340-5p	miR-340-5p inhibit STING expression to inhibit microglia activation and attenuat neuroinflammation	Primary neurons, microglia and C57BL/6 mice	(160)

It is noteworthy that both the knockout cGAS mice and the use of cGAS inhibitors appear to suppress the neuroinflammatory response in ischemic stroke. This strongly suggests that the cGAS-STING pathway can serve as a potential target for intervening in the neuroinflammatory response of ischemic stroke. However, given that the cGAS-STING pathway is a vital component of the innate immune system and exhibits significant expression in various immune cells, it is essential for the response to microbiota in homeostasis, thereby allowing the proper development of the adaptive immune system (161). When using cGAS KO mice as an ischemic stroke animal model, addressing potential extracerebral interferences becomes a concern (22, 24, 145). No studies have yet demonstrated whether cGAS KO induces numerous uncontrollable alterations in the immune system, potentially confounding ischemic stroke investigations. Targeting cGAS KO specifically in microglia might be pivotal in addressing this issue. The research generated mice lacking cGAS specifically in microglia by cross-breeding mice carrying cGAS‐floxed alleles (cGASf/+) with CX3CR1CreER transgenic mice (22). Using these mice, the specific importance of cGAS in stroke could be characterized.

### 4.4 Regulated cell death

The demise of neural cells is a deleterious outcome of various pathophysiological mechanisms in ischemic stroke. It has been substantiated that the RCD of neural cells directly impacts the enlargement of the ischemic penumbra and subsequent damage. A plethora of studies have been devoted to intervening in RCD to extend the thrombolytic window for the management of ischemic stroke, and some of these studies have demonstrated interactions between the cGAS-STING pathway and multiple RCDs (40, 134, 162, 163). However, the current understanding of the importance of cGAS-STING pathway in RCD processes such as ferroptosis and PANoptosis remains limited. The present section aims to enhance comprehension by synthesizing the existing knowledge on the cGAS-STING pathway.

#### 4.4.1 Ferroptosis

Ferroptosis is a newly identified form of non-apoptotic RCD characterized by the accumulation of lipid peroxides dependent on iron. It is morphologically and biochemically unique from other types of cell death. Ferroptosis is critical in the pathophysiology of tumors, renal failure, and ischemia/reperfusion injury (164). Senescent cells exhibit a reduced ability to scavenge iron ions, and ischemic injury exacerbates senescence, leading to the excessive accumulation of iron ions that ultimately triggers ferroptosis (165). Several studies employing MCAO models have demonstrated the significant therapeutic potential of ferroptosis inhibitors (166–168). It has been established that ferroptosis is closely associated with neuronal damage during stroke.

Polyunsaturated fatty acids (PUFAs) peroxidation within cellular membranes is a pivotal element in ferroptosis. Research has provided evidence that the STING protein modulates PUFA metabolism by impeding the function of delta-6 desaturase (Δ6D), a rate-limiting enzyme of fatty acid desaturase 2 (FADS2) (169). This inhibition results in the accumulation of PUFA derivatives. Current evidence suggests a feedback loop with negative regulation between STING and PUFA metabolism, as PUFAs can impede STING activation (131). Excessive ROS can directly impact STING activity, which oxidizes cysteine 147 of the STING protein, ultimately inhibiting its aggregation and downstream signaling. The preservation of cellular redox homeostasis necessitates the presence of GPX4 (131). When GPX4 is deficient, cellular lipid peroxidation occurs, leading to carbonylation of STING at C88 and hindering its transit from the ER to the Golgi apparatus (132). The activation of STING and its downstream transcription factors is impeded when cellular antioxidant capacity is diminished or ROS production is augmented. On the other hand, STING itself can modulate lipid peroxidation, highlighting its role in maintaining metabolic homeostasis. However, over-activation of STING may disrupt PUFA metabolism and contribute to ferroptosis.

The cGAS-STING pathway activation has been associated with excess iron deposition in cells. Previous studies have shown that iron treatment increases the expression of cGAS, STING, and their downstream targets (170). As illustrated in section 4.1, during the early phases of ischemia-reperfusion (I/R), NCOA4 exacerbates intracellular free Fe2+ accumulation by promoting ferritinophagy. This increase in the labile iron pool (LIP) subsequently amplifies cellular membrane damage to polyunsaturated fatty acids (PUFA) via the Fenton reaction (129). The expression of NCOA4 is also regulated by the upstream cGAS-STING pathway, further intensifying the ischemic injury induced by ferritinophagy (130). Furthermore, it has pinpointed Q237, E316, and S322 in the CBD domain of STING as crucial binding sites that interact with the coiled-coil domain of NCOA4 (171). Numerous investigations have demonstrated that NCOA4 serves as an upstream mechanism to elicit ferroptosis by controlling intracellular levels of free iron and the production of cellular ROS (172, 173). Notably, STING stimulates NCOA4 to induce ferritinophagy, underscoring the crosstalk between the cGAS-STING pathway and ferroptosis in regulating intracellular iron homeostasis.

Reduction of antioxidant capacity and initiation of ferroptosis is a consequence of inhibiting the cystine/glutamate antiporter or system Xc^−^. Following an ischemic stroke, the release of DAMPs from necrotic cells and the impaired BBB result in microglia activation and infiltration of peripheral immune cells (174). The cGAS-STING pathway is reportedly involved in the immune response by releasing pro-inflammatory cytokines, which promotes immune cell infiltration (23). Downstream IFN-1 induces the proliferation and survival of CD8^+^ T cells by activating the JAK-STAT3 and MAPK pathways (175) During the initial phases of ischemic stroke, the infiltration of CD8^+^ T cells in the ischemic penumbra is minimal; however, it gradually increases as time progresses. These cells predominantly infiltrate the center of the cerebral infarct, and their abundance is directly correlated with the size of the infarct (176, 177). Mechanistically, the activation of CD8^+^ T cells results in the release of IFN-γ, which regulates the expression of two subunits of the cystine/glutamate antiporter, namely solute carrier family 3 members 2 (SLC3A2) and solute carrier family 31 member 1 (SLC7A11) (178). When the function of System Xc- is inhibited, there is a significant reduction in cellular glutathione levels. Given that GPX4 utilizes glutathione as an indispensable cofactor for its enzymatic activity, this results in the indirect inhibition of GPX4 (179).

**Figure 4:**
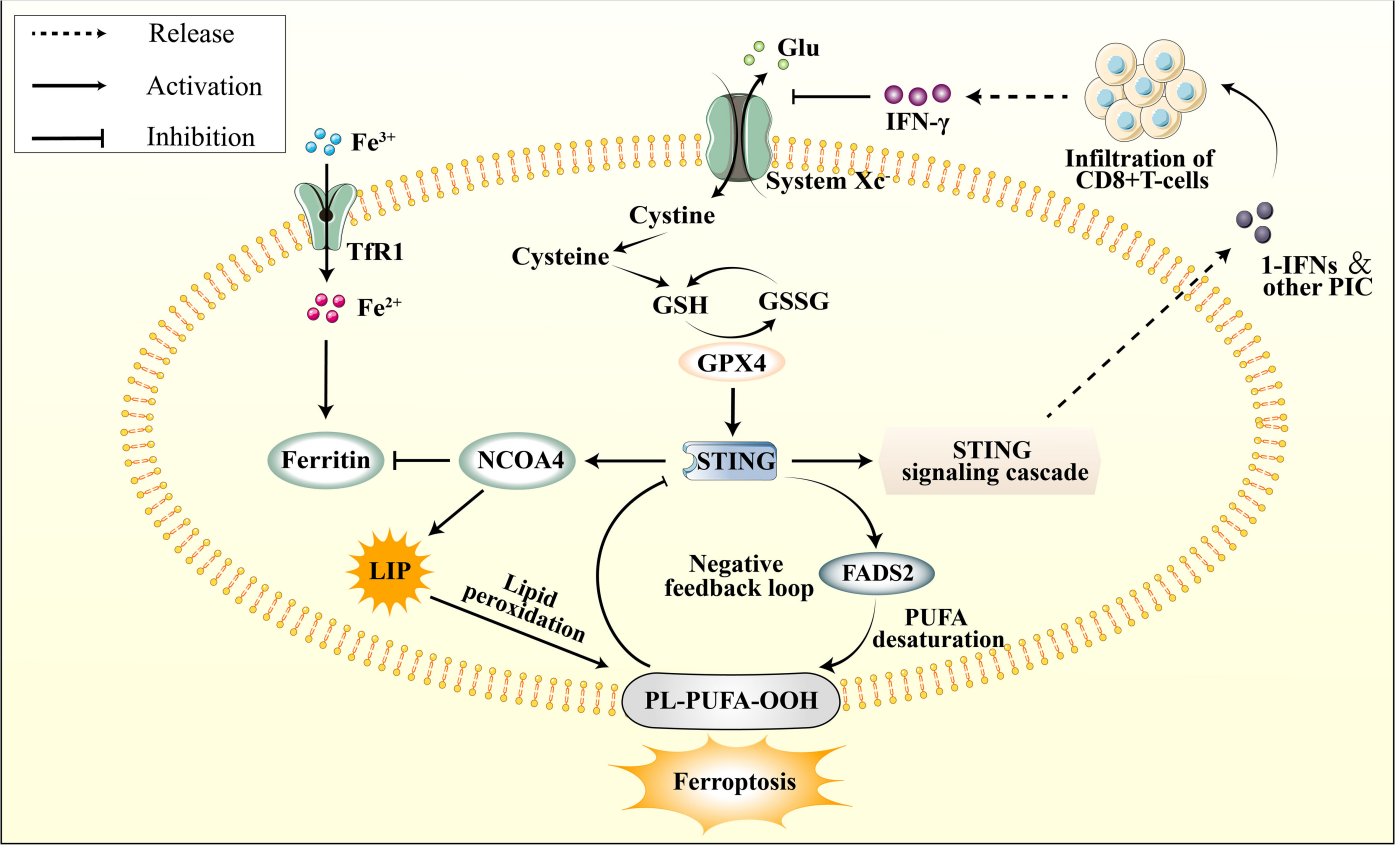
The connection between cGAS–STING pathway and ferroptosis. The cGAS-STING pathway is subject to physiological negative regulation by ferroptosis in multiple respects, while its excessive activation is implicated in the initiation of ferroptosis. STING promotes the upregulation of NCOA4, subsequently inhibiting Ferritin, leading to an elevation in the LIP. This results in lipid peroxidation of the cellular membrane’s PUFA, thereby inducing ferroptos0069s. Additionally, STING promotes the activation of FADS2, which enhances PUFA levels, thereby providing a substrate for lipid peroxidation. However, PUFA can impede STING activation. The infiltration of peripheral CD8+ T cells modulates the function of system Xc- through IFN-γ signaling. This leads to a depletion of glutathione, indirectly inhibiting GPX4 activity, subsequently attenuating GPX4’s promotive effect on downstream STING.

By far, the precise mechanism by which the cGAS-STING pathway is implicated in ferroptosis during ischemic stroke remains unclear. However, it has been established that excessive activation of the cGAS-STING pathway during cerebral ischemia leads to NCOA4 overexpression, resulting in iron accumulation in neuronal cells [105]. The elevation of intracellular free iron ions induced by the cGAS-STING pathway is a critical contributor to ferroptosis. Furthermore, current evidence suggests that the cGAS-STING pathway is linked to PUFA peroxidation, impaired GPX4 antioxidant function, and system Xc^−^ dysfunction (**Figure 4**). It is highly conceivable that the cGAS-STING pathway involves several aspects of ferroptosis in ischemic stroke. Furthermore, considering the predominant distribution of cGAS in immune cells, this could potentially represent an innovative mechanism for immunogenic cell death (ICD).

#### 4.4.2 PANoptosis

PANoptosis is a novel form of RCD associated with inflammation and is mediated by the PANoptosome complex. Despite sharing features of pyroptosis, apoptosis, and necroptosis, PANoptosis cannot be exclusively categorized as any of these RCD types (180). Studies have demonstrated that innate immunity can concurrently regulate pyroptosis, apoptosis, and necroptosis through the PANoptosome complex, in which Z-DNA binding protein 1 (ZBP1) with Zα molecules is crucial (39). The cGAS-STING pathway, a constituent of the innate immune system, can initiate various forms of RCD, such as pyroptosis, apoptosis, and necroptosis, suggesting that the cGAS-STING pathway may regulate PANoptosis (22, 56, 181, 182). Additionally, a comprehensive analysis has verified the occurrence of PANoptosis in ischemic brain injury, indicating its potential as a therapeutic target (183).

Pyroptosis is a type of RCD characterized by the activation of the inflammasome, the maturation of pro-inflammatory cytokines such as IL-1β and IL-18, and the involvement of the Gasdermin family proteins that cause damage to the cell membrane (184). Recent findings suggest that pyroptosis in microglial cells is closely associated with the development of ischemic brain injury (147, 185, 186). Specifically, increased cerebral ischemia-induced cytoplasmic dsDNA activates the cGAS-STING pathway, and inhibition of cGAS leads to the downregulation of inflammasome AIM2, caspase-1, gasdermin D, IL-1β, and IL-18 (22). Although the precise mechanism by which the cGAS-STING pathway and AIM2 participate in the inflammatory reaction during ischemic stroke remains ambiguous, the interaction of downstream NF‐κB with GSDMD or type I IFN with AIM2 may be involved. Additionally, another study on neuroinflammation demonstrated that the administration of the cGAS inhibitor RU.521 impeded the activation of the cGAS-STING pathway and diminished the expression of caspase-1, GSDMD, and inflammasome NLRP3, thereby mitigating microglial pyroptosis and reducing the neuroinflammatory response (27). In contrast, the activation of the STING pathway through ADU-S100 promoted microglial activation and neuroinflammatory responses, leading to the upregulation of caspase-1 and GSDMD and exacerbating pyroptosis-related activation (187). Treated with the STING antagonist c-176 yielded the opposite effects. These studies collectively indicate that the cGAS-STING pathway is pivotal in modulating microglial pyroptosis. Additionally, the activation of the cGAS-STING pathway induces pyroptosis in other ischemic organs, including the liver and intestine (188, 189).

Apoptosis represents the predominant form of RCD. Within ischemic regions, apoptosis is triggered by the accumulation of extracellular glutamate, which results from the compromised uptake of this excitatory neurotransmitter by ischemic neurons (190). *In vitro* experiments on HT22 cells exposed to oxygen-glucose deprivation and BV2 microglial cells stimulated with cell culture supernatant demonstrated microglial polarization and heightened cGAS, STING, and pro-inflammatory cytokines (23). However, applying cGAS inhibitors significantly impeded these outcomes and effectively mitigated neuronal apoptosis in MCAO mice. Interestingly, the cGAS-STING pathway is inhibited by apoptosis, as evidenced by the heightened TBK1 phosphorylation and IFNα expression observed in caspase-9 knockout or caspase-3/-7 double knockout mice and cells (191). A similar increase in IFN-1 expression was noted in caspase-9-deficient mouse embryonic fibroblasts (32). This phenomenon may be attributed to the degradation of cytoplasmic mtDNA mediated by Caspases (56). Moreover, activated caspase-3 can reportedly cleave cGAS and IRF3 to prevent excessive IFN production (192).

Necroptosis is a form of RCD dependent on RIPK1 and involves the formation of a complex with downstream RIPK3 to phosphorylate and activate mixed lineage kinase domain-like (MLKL), ultimately resulting in cell membrane rupture (193). The subsequent release of various DAMPs triggers an innate immune response (194). A study on sepsis-associated encephalopathy indicated that STING expression in hippocampal neurons is elevated and interacts with PERK, a significant factor of ER stress, to promote p-RIPK3/RIPK3 expression in damaged neurons (181). This suggests that modulation of the PERK-STING-RIPK3 pathway may have neuroprotective effects by reducing neuroinflammation. Additionally, increased levels of dsDNA in damaged cells have been observed to activate the cGAS-STING pathway in various ischemic diseases, leading to downstream IFNs and other pro-inflammatory cytokines that can mediate necroptosis, such as intestinal and renal ischemia (195, 196). Furthermore, RIP3 interacts with inner mitochondrial proteins to facilitate their degradation, leading to increased damage to mitochondria and the release of mtDNA, activation of the cGAS-STING/p65 pathway, and amplification of the damage (196). Over the past few years, the involvement of necroptosis in cerebral ischemic injury has gained widespread recognition (197–200), implying that the cGAS-STING pathway may serve as a new mechanism for mediating necroptosis in neurons and that inhibiting its activation could provide neuroprotection.

**Figure 5:**
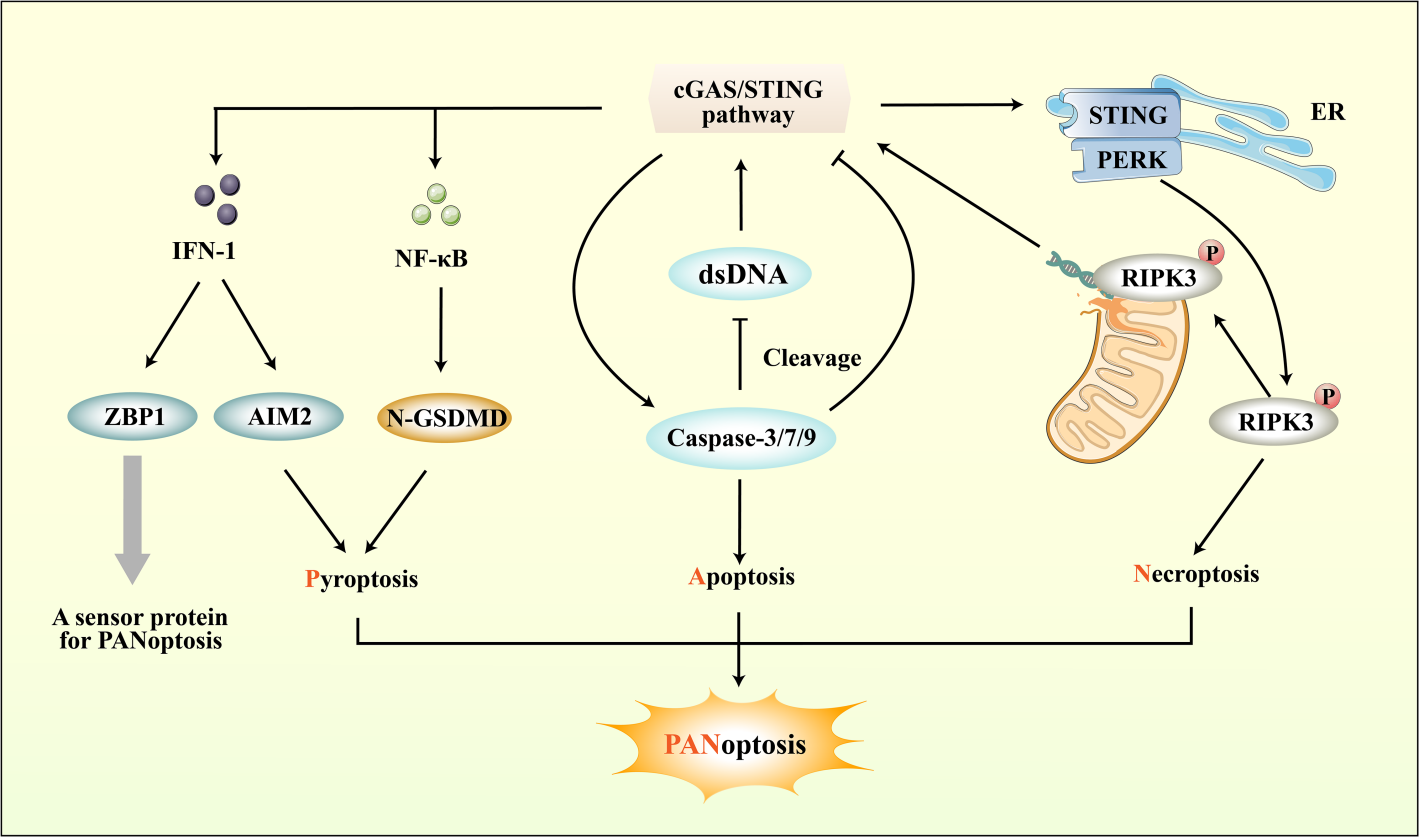
The connection between cGAS-STING pathway and PANoptosis. ZBP1, a sensor protein for PANoptosis, can be induced by the IFN-1 downstream of the cGAS-STING pathway. Importantly, the cGAS-STING pathway not only directly regulates PANoptosis through IFN-1, but also modulates pyroptosis, apoptosis, and necroptosis respectively to regulate PANoptosis. While the cGAS-STING pathway downstream of pro-inflammatory cytokines can promote pyroptosis, it can also regulate Caspase protein-induced apoptosis. Moreover, cGAS-STING mediates necroptosis by interacting with PERK and promoting downstream RIPK phosphorylation.

Through the integration of prior research on cerebral ischemic injury and the mechanisms of RCD, including pyroptosis, apoptosis, and necroptosis, it has been determined that these processes can co-occur and contribute to neuroinflammation (183). Consequently, PANoptosis as a novel form of RCD raises the possibility of its involvement in ischemic stroke. While its impact on CNS injury has received less scrutiny, recent investigations have indicated the potential for interplay between PANoptosis and cerebral ischemic injury via ceRNA regulatory networks (201). The cGAS-STING pathway can directly upregulate the expression of ZBP-1, a sensor protein for PANoptosis, through downstream IFN-1, apart from its direct role in pyroptosis, apoptosis, and necroptosis (202). Recent research has revealed that STING agonists can also lead to the upregulation of other markers associated with necroptosis and apoptosis, such as MLKL and caspase-3, as well as inflammasome NLRP3 and AIM2 (203). The above studies overlap in their assertion that the cGAS-STING pathway can regulate PANoptosis through multiple pathways (**Figure 5**).

## 5 Targeting cGAS-STING pathway to mitigate side effects of thrombolytic therapy against ischemic stroke

Thrombolytic therapy has the potential to dissolve blood clots and re-establish blood flow to ischemic organs, including the brain, thereby serving as an efficacious intervention for neurological impairment and improving survival in individuals with ischemic stroke. Nevertheless, this therapeutic approach is linked to a heightened risk of HT, with documented incidence rates ranging from 2.4% to 10% within the initial 24-36 hours following thrombolysis (204). HT poses a significant obstacle to reperfusion therapy and adversely affects patient prognosis. Activation of the cGAS-STING pathway has been shown to affect the development of HT after intravenous thrombolysis. Therefore, it is recommended to consider the modulation of cGAS sensitivity to dsDNA or the inhibition of the cGAS-STING pathway as potential strategies to mitigate the side effects of thrombolysis.

### 5.1 Hemorrhagic transformation

**Figure 6:**
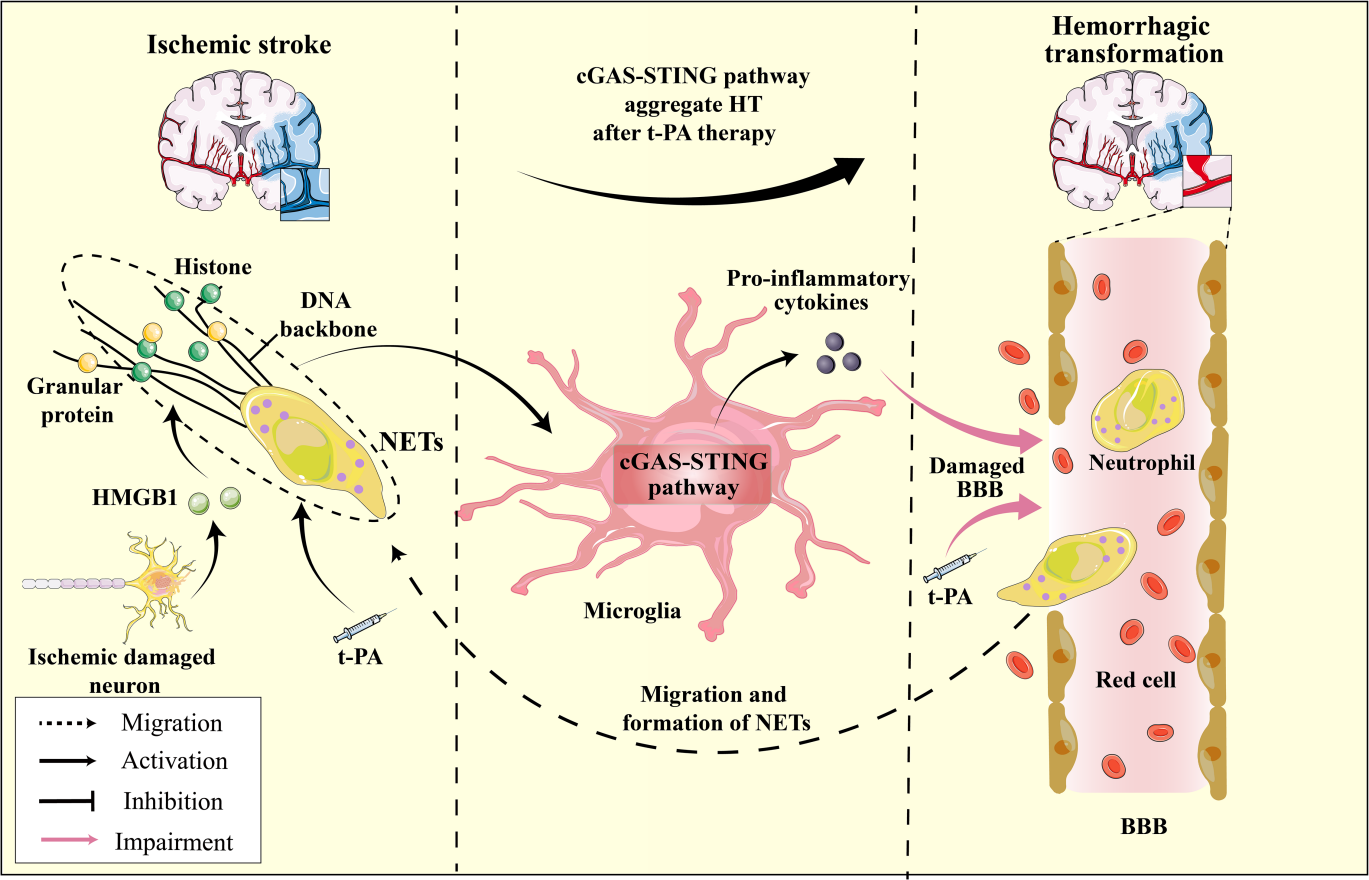
The HMGB1-NETs-cGAS-STING pathway serves as a mediator for hemorrhagic transformation: A Hypothesis. In ischemic stroke, ischemic neurons release HMGB1, enhancing cGAS’s affinity for the NETs scaffold, which in turn activates the microglial cGAS-STING pathway and induces pro-inflammatory cytokine production. t-PA treatment for ischemic stroke further promotes neutrophil degranulation and NET formation, exacerbating the activation of the microglial cGAS-STING pathway. The released pro-inflammatory cytokines, in conjunction with t-PA, compromise the BBB, increasing its permeability, leading to brain hemorrhage. Furthermore, as neutrophils migrate to the ischemic region and the continuous formation of NETs ensues, this process intensifies, resulting in HT during t-PA treatment for ischemic stroke.

NETs are reticular complexes composed of dsDNA, histones, and granular proteins released by activated neutrophils (205). The administration of t-PA for thrombolysis can trigger neutrophil degranulation, resulting in the formation of NETs, which can cause hemorrhagic transformation and resistance to thrombolysis (206). Furthermore, t-PA can compromise vascular integrity and BBB function, exacerbating neutrophil infiltration in the ischemic region and penumbra (207, 208) (**Figure 6**). DNase I has been shown to disrupt NETs, mitigate cerebral ischemic injury, and alleviate the adverse effects of t-PA thrombolysis (209, 210). DNase I has demonstrated efficacy in mitigating ischemic damage during thrombolysis for ischemic stroke and exhibiting potential for targeting NETs in other ailments, including myocardial and experimental hindlimb I/R injury (211, 212). Studies have revealed that target NETs via DNase I can inhibit the activation of cGAS-STING pathway, thereby reducing cerebral ischemic damage (24). Indeed, it is widely thought that the upregulation of pro-inflammatory cytokines are not directly facilitated by microglia, but rather by the infiltration of neutrophils and subsequent release of substantial quantities of NETs (213). These findings imply that the activation of the cGAS-STING pathway is closely linked to NETs and may represent a pivotal molecular mechanism underlying the interplay between microglia and neutrophils. Recent research has indicated that the administration of t-PA leads to the promotion of cGAS expression in activated microglia and the promotion of downstream signals such as STING, TBK1, pIRF3, and IFN-1 expression (24). Additionally, the cGAS-/- can restore the integrity of the BBB that has been disrupted by t-PA, which reduces the infiltration of neutrophils and the formation of NETs (24). The activation of the microglia cGAS-STING pathway by the DNA backbone in NETs promotes neutrophils migration and increased NET formation, creating a positive regulatory loop between NETs and the cGAS-STING pathway (**Figure 6**). This novel mechanism may contribute to the adverse effects of thrombolytic therapy. Furthermore, the activation of the microglial cGAS-STING pathway is central to the entire process.

Moreover, the modulation of cGAS responsiveness to dsDNA may heighten and exacerbate susceptibility to HT. Research has indicated that the sensitivity of h-cGAS to dsDNA is contingent upon the length of the DNA molecule. Specifically, a DNA length of approximately 45 base pairs constitutes a threshold below which cGAS activation is not observed (10). It has been postulated that certain intracellular factors, such as HMGB proteins 1/2 and TFAM, can augment DNA recognition by cGAS. These proteins facilitate the proper arrangement of DNA by forming U-turns, thereby amplifying the activation and sensitivity of cGAS (214, 215). Indeed, the sensitivity and activity of cGAS to dsDNA can be increased by up to 25-fold with adequate levels of human HMGB1 (53). HMGB1, a DAMP, can activate various PRRs, including Toll-like receptors, matrix metalloproteinase enzymes, and receptors for advanced glycation end products (RAGEs), leading to cerebral ischemia and neuroinflammatory damage (216). HMGB1 is initially released passively from dying neurons and subsequently secreted actively by infiltrating microglia/macrophages during the early stages of stroke (217). Despite not serving as a ligand for cGAS, HMGB1 facilitates the formation of U-turns in DNA, thereby promoting cGAS-mediated innate immunity, which may prove crucial in the context of the pronounced HMGB1-induced injury observed following stroke. The HMGB1 binding heptapeptide (HBHP) represents an inhibitor of HMGB1 that effectively mitigates damage to the BBB and the incidence of HT after t-PA thrombolytic therapy (218). The potential modulation of cGAS sensitivity to dsDNA by HMGB1 presents a novel perspective for pharmacological intervention in ischemic stroke through HMGB1 antagonists or monoclonal antibodies.

It has been established that the upregulation of HMGB1 is crucial to induce H3cit production in both central and peripheral neutrophils in an MCAO model (219). This finding implies that HMGB1 may serve as an upstream regulator of NETs, facilitating their generation, while concurrently modulating the affinity of NETs for their downstream receptor cGAS and mediating the activation of the cGAS-STING pathway. In essence, a direct correlation exists between HMGB1, NETs, and cGAS, and the HMGB1-NETs-cGAS molecular pathway may function as a “catalytic mediator” for the activation of the cGAS-STING pathway (**Figure 6**).

### 5.2 Nevascularization

Neovascularization is an innate reparative mechanism of the central nervous system in response to brain injury. Impaired neovascularization exacerbates cerebral ischemic injury, thereby hindering thrombolytic therapy (220, 221). It has been shown that levels of circulating DNA in the infarcted region are significantly elevated, which may be attributed to incomplete development and high permeability of neovascularization (222). The augmented levels of free DNA can directly activate the DNA sensor cGAS. Research has demonstrated that MCAO mice exhibit significantly upregulated STING, TBK1, and IRF3 expression (222). Notably, silencing STING or blocking IFNAR function in mice enhanced vascular regeneration and repair (213). These findings imply that IFN-1 is closely associated with revascularization, and the reduction of DNA levels or suppression of the cGAS-STING pathway may facilitate neovascularization.

## 6 Conclusion and perspective

The application of immunomodulatory therapy for ischemic stroke has become a research hotspot in recent years (223–227) (**Table 2**). It is now understood that the innate immune system is promptly activated following ischemia, which aids in eliminating necrotic cells and promotes tissue restoration, while also playing a significant role in initiating and intensifying post-stroke inflammation. While most research has concentrated on the immune system’s function after stroke, a growing body of evidence indicates that inflammation and immune activation preceding brain injury can impact stroke susceptibility and prognosis. In Western countries, the prevalence of co-morbidities, including obesity, hypertension, and diabetes, is high, resulting in the widespread presence of pre-existing chronic “low-grade” systemic inflammation in stroke pathophysiology. The systemic inflammatory response and immune dysregulations associated with stroke can significantly impact brain injury, recovery, and outcome. Although the modulation of inflammatory and immune responses in the brain can directly influence the clinical presentation and outcome of stroke, there are limited proven treatment options. Consequently, a rational approach to managing ischemic stroke may involve the safe and effective modulation of the innate immune system of CNS.

**Table 2:** Immunomodulatory therapy in ischemic stroke.

Drug	Mechanism	Result	Type of Research	Reference
Minocycline	Minocycline regulated microglial M1/M2 polarization through STAT1/STAT6 pathways.	Reducing cytokine production, promoting neuronal survival, and neurological functional recovery.	Experimental research.	(223)
Recombinant human IL-1 receptor antagonist (rhIL-1Ra)	Treatment with IL-1Ra reverses peripheral innate immune suppression, which is associated with attenuated cortisol production.	TNF-α, IL-1β, IL-6, IL-8 and IL-10 by LPS was significantly reduced	Clinical trials	(224)
Natalizumab	Preventing cell adhesion molecules from entering the brain and inhibiting immune cells from entering the CNS.	Improving the prespecified secondary and tertiary endpoints as measured by mRS, BI, SIS-16, and MoCA scores.	randomized, placebo-controlled trial	(225)
Fingolimod	Limiting the lesion expansion by suppression of reperfusion injury	Combination therapy of fingolimod and alteplase was well tolerated, attenuated reperfusion injury and improved clinical outcomes in AIS patients	Clinical trials	(226)
Edaravone dexborneol	Eliminating lipid peroxides and hydroxyl radicals.	Administering within 48 hours after AIS results in favorable functional outcomes at 90 days, particularly in female patients.	Randomized, double-blind, comparative trial	(227)

This review centers on the potential application of the cGAS-STING pathway as an effective approach to address the issue from pre- to post-ischemic stroke. Analogous to other innate immune pathways, the cGAS-STING pathway has a mixed-blessing effect. It promotes microglia polarization and neuroinflammation, which exacerbate ischemic stroke injury and expand the extent of damage while enhancing host defense mechanisms against ischemic injury. We provide a comprehensive and multidimensional systematic review of ischemic stroke, covering the pre-onset to treatment, emphasizing the inflammatory response triggered by the cGAS-STING pathway. Within the existing research, we introduce three innovative concepts. Firstly, metabolic dysfunction can induce co-activation of both peripheral and central cGAS-STING pathways, thereby increasing susceptibility to stroke. Factors such as Ang II, HFD, and fatty acids in daily food intake can activate the cGAS-STING pathway in microglia, leading to neuroinflammation and vulnerability to ischemic stroke. Secondly, the etiology of ischemic stroke-induced ICD has been attributed to peripheral immune cells, specifically CD11b^+^CD45^+^cells (228). Conversely, RCD, governed by cGAS-mediated microglia, may represent a novel form of ICD instigated by intrinsic immune cells within the brain (229). Our investigation delved into the interplay between upstream or downstream regulators of cGAS-STING and ferroptosis and PANoptosis. Interestingly, the cGAS-STING pathway reportedly governs ferroptosis within the ischemic penumbra by heightening intracellular iron concentrations and diminishing cellular antioxidant capability following the onset of cerebral ischemia. Furthermore, we suggest that IFN-1, produced by the cGAS-STING pathway, could facilitate the activation of ZBP-1 to prompt PANoptosis. Thirdly, the side effects of thrombolytic therapy in ischemic stroke may be associated with the regulation of NETs affinity for cGAS by HMGB1 released from dying neurons and the direct promotion of NETs by HMGB1. We posit that the HMGB1-NETs-cGAS-STING positive feedback loop constitutes a pivotal molecular interplay involving neurons, vascular endothelial cells, neutrophils, and microglia, which serves as a novel mechanism underlying the HT following thrombolysis in ischemic stroke. Insights from this review provide valuable insights for developing complementary clinical t-PA thrombolytic therapies, such as HMGB1 monoclonal antibodies, DNase I, and cGAS-STING inhibitors.

From 2020 onward, researchers began to focus on and explore the importance of the cGAS-STING pathway in ischemic stroke (22). Although research on this pathway and its involvement in ischemic stroke is still in its infancy, progress has been made. For instance, researchers have employed nanomaterials to penetrate the BBB and target the modulation of cGAS-STING pathway activity in the ischemic zone (25). Certain natural compounds have been discovered to effectively inhibit the cGAS-STING pathway (230–232). Moreover, recent research has uncovered that nuclear cGAS remains inactive in the presence of chromatin, indicating the existence of inherent cellular mechanisms and active agents that directly inhibit cGAS activity (51, 233). From a safety perspective, since all molecules in the cGAS-STING pathway are upstream of IFN-I, targeting this pathway is expected to have a lesser effect on host defenses than blocking the IFN-I receptor. These discoveries provide practical and theoretical bases for further exploration of the regulation of the cGAS-STING pathway as a potential intervention for ischemic stroke. However, many questions remain unanswered, such as whether cGAS is regulated by other small molecules, whether it has consistent sensitivity to different types of DNA, whether cGAS recognizes any other ligands besides dsDNA, and whether there is crosstalk between the cGAS-STING pathway and other immune pathways activated by the release of DAMPs/PAMPs from ischemic neuronal cells, like TLRs or NLRs. Furthermore, the specific regulation of TBK1 and NF-κB as downstream transcription factors of the cGAS-STING pathway remains unclear. Further investigation is needed to elucidate the precise mechanisms and factors involved in regulating the activity of TBK1 and NF-κB within this pathway. Besides, it is important to consider the inflammatory protective effect of the cGAS-STING pathway in the physiological state. Crucially, the exact mechanisms through which the cGAS-STING pathway modulates pro-inflammatory and anti-inflammatory responses in microglia remain elusive. Given its ‘double-edged sword’ nature, whether the cGAS-STING pathway is intricately involved in regulating the delicate balance between pro-inflammatory attributes and neuroprotective characteristics of various microglial subpopulations warrants further exploration (234). Subsequent research should concentrate on the precise and rational modulation of this pathway while circumventing excessive inflammatory reactions. While human clinical trials have been disappointing thus far, it is highly conceivable that h-cGAS and m-cGAS are involved in different processes, given that they only share 60% amino acid identity (68). Therefore, drug screens targeting h-cGAS specifically could be valuable. Targeting this pathway will likely be a therapeutic and preventative strategy for ischemic stroke.

## Author contributions

XM: Conceptualization, Data curation, Software, Visualization, Writing – original draft. DX: Conceptualization, Formal Analysis, Software, Writing – original draft. RS: Conceptualization, Methodology, Writing – original draft. DL: Conceptualization, Software, Visualization, Writing – original draft. JG: Supervision, Writing – review & editing. ZM: Conceptualization, Supervision, Writing – review & editing.

## Glossary

**Table:**

AMPK	AMP-activated protein kinase
Ang II	Angiotensin II
ApoE	Apolipoprotein E
AS	Atherosclerosis
A-T	Ataxia‐Telangiectasia
ATF6	Activating transcription factor-6
BAF	Barrier-to-autointegration factor 1
BBB	Blood-brain barrier
BP	Blood pressure
CCL-2	Chemokines (C-C motif) ligand-2
CCL-3	Chemokines (C-C motif) ligand-3
cGAMP	Cyclic GMP-AMP;cGAS, Cyclic GMP-AMP synthase
CI/RI	Cerebral ischemia/reperfusion injury
CVST	Cerebral venous sinus thrombosis
DAMPs	Damage-associated molecular patterns
DNase	Deoxyribonuclease;DsbA-L, Disulfide-bond A oxidoreductase-like protein
dsDNA	Double-stranded DNA
EndMT	Endothelial-to-mesenchymal transition
ER	Endoplasmic reticulum
FADS2	Fatty acid desaturase 2
FTO	fat mass and obesity-associated protein
GPX4	Glutathione peroxidase 4
HBHP	HMGB1 binding heptapeptide
HDAC3	Histone deacetylase 3
HFD	High-fat diet
HMGB1	High-mobility group box 1
HT	Hemorrhagic transformation
ICD	Immunogenic cell death
IFN	Interferon
IKK	Inhibitor of kappa B kinase
iNOS	Inducible nitric oxide synthase
IRE1α	Inositol-requiring enzyme 1α
IRF-3	Interferon regulatory factor 3
ISGs	IFN-stimulated genes
LINE-1	Long-interspersed element-1
LIP	Labile iron pool
MCAO	Middle cerebral artery occlusion
m6A	N6-Methyladenosine
MLKL	Mixed lineage kinase domain-like
MST1	Mammalian Ste20-like kinases 1
mtDNA	Mitochondrial DNA
NCOA4	Nuclear receptor coactivator 4
NETs	Neutrophil extracellular traps
NF-κB	Nuclear factor kappa B
NLRs	NOD-like receptors
NO	Nitric oxide
NTase	Nucleotidyl transferase
nuDNA	Nuclear DNA
PA	Palmitic acid
PAMPs	Pathogen-associated molecular patterns
PERK	PKR-like ER-resident kinase
PRRs	Pattern recognition receptors
PUFAs	Polyunsaturated fatty acids
RAGEs	Receptors for advanced glycation end products
RAS	Renin-angiotensin system
RCD	Regulated cell death
RLHs	RIG-I-like helicases
ROS	Reactive oxygen species
SASP	Senescence-associated secretory phenotype
SLC3A2	Solute carrier family 3 member 2
SLC7A11	Solute carrier family 31 member 11
STIM1	Stromal interaction molecule 1
STING	Stimulator of interferon genes
TBK1	TANK-binding kinase 1
TFAM	Mitochondrial transcription factor A
TGF-b	Transforming growth factor b
TLRs	Toll-like receptors
t-PA	Tissue-type plasminogen activator
TREX1	Three prime repair exonuclease 1
UPR	Unfolded protein response
YAP	Hippo-Yes-associated protein
ZBP1	Z-DNA binding protein 1
Δ6D	Delta-6 desaturase
